# Adjuvant Radiation Survival Benefits in Patients with Stage 1B Rectal Cancer: A Population-based Study from the Surveillance Epidemiology and End Result Database (1973–2010)

**DOI:** 10.7759/cureus.6299

**Published:** 2019-12-05

**Authors:** Abdul Waheed, Frederick D Cason

**Affiliations:** 1 Surgery, Bolan Medical College, Quetta, PAK; 2 Surgery, San Joaquin General Hospital Program, French Camp, USA

**Keywords:** stage 1b rectal cancer, t2n0, adjuvant radiation, surgery, tme, tem

## Abstract

Introduction

Rectal cancer remains a leading cause of cancer morbidity and mortality in the United States. Currently, total mesorectal excision (TME) is the standard therapy for patients with T2N0 (stage IB) rectal cancer. Whether adjuvant radiation therapy provides a survival benefit to these patients or exposes them to unnecessary toxicity remains controversial and unproven to date. This study examined a large cohort of Stage 1B rectal cancer patients who underwent surgical resection and received adjuvant radiation in order to determine the demographic, clinical, and pathologic factors impacting prognosis and survival.

Methods

Demographic and clinical data on 4,054 Stage 1B rectal cancer patients were abstracted from the Surveillance Epidemiology and End Result (SEER) database (1973-2010). Statistical analysis was performed with SPSS v20.0 software (IBM Corp., Armonk, NY) using the chi-square test, paired t-test, multivariate analysis, and Kaplan-Meier functions.

Results

Among 4,054 patients with stage IB rectal cancer, 2,364 (58.3%) had surgery only, 1,477 (36.4%) received combination surgery and radiation (CSR), 139 (3.4%) received radiation only, and 74 (1.8%) received no therapy. Most stage IB patients in the surgery only and CSR groups were male (65.8 and 64%) and Caucasian (78.2% and 74.2%), p<0.001. Patients receiving CSR were younger than those undergoing surgery alone (63 vs. 69 years, p<0.001). More tumors in the CSR group were 2-4 cm (53.6%), followed by > 4 cm (24%), while fewer were <cm (22.4%). Histologically, most of the tumors in the CSR group were moderately differentiated (83.5%) and adenocarcinoma NOS (95.5%), followed by poorly (9.3%) and mucinous adenocarcinoma (4.5%), well-differentiated (6.8%), and undifferentiated (0.4%). Overall survival was prolonged in the CSR group compared to the surgery-only group (5.85 years vs. 5.44 years, p<0.001), although cancer-specific survival did not differ (6.33 years vs. 6.42 years, p=0.143). Multivariate analysis identified age>60 (OR 2.4), poorly differentiated (OR 1.7) or undifferentiated grade (OR 2.6), and tumor size >2 cm (OR 1.5) as independently associated with increased mortality in the CSR group (p<0.05) while female gender conferred a survival advantage (OR 0.8), p<0.01.

Conclusions

In the current cohort, CSR was utilized most often in young male Caucasian patients presenting with less advanced disease as compared to other treatment groups. The overall survival is prolonged and overall mortality is lower in patients receiving CSR; however, increased cancer-related mortality with the use of CSR implies that survival benefits may be attributable to favorable non-tumor-related factors such as age, gender, and race. CSR should not replace surgery alone as the standard of care for all Stage IB rectal cancer patients at this time. However, all T2N0 rectal cancer patients should be enrolled in randomized control trials to allow for more defined multimodality management to optimize clinical outcomes for these patients.

## Introduction

Rectal cancer is the third most frequent cancer diagnosis for both men and women in the United States (US) [1]. In the US, more than 42,000 individuals are diagnosed with rectal cancer annually and more than 8,500 die each year [2]. An estimated 39,610 cases of rectal cancer are expected to be diagnosed in 2015 [3]. Preoperative chemoradiation is the standard therapy for patients with T3/T4 or N1 rectal cancer while primary surgical treatment with total mesorectal excision (TME) has historically been the mainstay of management for T2N0 rectal cancer [4]. Whether these patients benefit from adjuvant radiation therapy or merely endure unnecessary toxicities remains controversial and unproven to date.

Currently, no consensus exists on the best course of management for T2N0 rectal cancers and studies are limited. Also, demographics, pathologic, and clinical factors influencing prognosis and survival in T2N0 rectal cancer patients receiving adjuvant radiation are not well-understood. The current study examines a large cohort of T2N0 rectal cancer patients from the Surveillance, Epidemiology, and End Results (SEER) database to determine demographic, clinical, and pathologic factors impacting prognosis and survival in patients receiving adjuvant radiation therapy.

## Materials and methods

Data for the current study were extracted from the Surveillance, Epidemiology, and End Result (SEER) database, which is part of the National Cancer Institute, between 1973 and 2010. SEER Stat software version 8.0.4 was utilized to extract data from 18 SEER registries (Alaska Native Tumor Registry, Arizona Indians, Cherokee Nation, Greater Bay Area Cancer Registry, Seattle-Puget Sound, Iowa, Connecticut, Detroit, Georgia Center for Cancer Statistics, Greater California, Los Angeles, New Jersey, Hawaii, Kentucky, Louisiana, New Mexico, and Utah). Almost 4,054 patients with histologically confirmed atypical T2N0 (Stage 1B) rectal cancer were identified and exported to IBM SPSS® v20.2 (Armonk, NY). The 4,054 patients with a primary diagnosis of T2N0 rectal cancer were identified to form the final study cohort using the SEER International Classification of Disease for Oncology (ICD-O-3) codes C20.1.

The demographic and clinical data extracted included various age groups, gender distribution and frequency, the racial makeup of the cohort, the tumor stage and its size, primary tumor site, and the type of treatment. All patients with in-situ cancers, those tumors that had a nonspecific site of tumor origin, and those in patients in whom the histological confirmation of the disease was not available were excluded from the final cohort. The endpoints examined included overall survival, cancer-specific survival, overall mortality, and cancer-specific mortality. Categorical variables were compared using the chi-square test and continuous variables were compared using the student t-test and analysis of variance (ANOVA). The “backward Wald” method was performed to calculate odds ratios (OR) for the multivariate analysis and to determine the independent factors affecting the patient’s survival. The Kaplan-Meier analysis was used to compare long-term actuarial survival between groups. Statistical significance was accepted at the level of p<0.05.

## Results

Incidence data

A total of 4,054 cases of T2N0 rectal cancer were reported in the SEER database between 1973 and 2010. Of these, 58.3% (N=2,364) received surgery alone, 36.4% (N=1,477) patients received a combination of surgery and radiation (CSR), 3.4% (N=139) of patients received radiation alone, and 1.8% (N=74) patients received no treatment (Table 1).

**Table 1:** Demographic Profiles of 4,054 Patients with T2N0 Rectal Cancer from the Surveillance, Epidemiology, and End Results (SEER) Database, 1973-2010 Abbreviations; N = number; * data presented for patients with available information only

T2 NO Rectal Cancer		Total		No Therapy		Surgery Only		Radiation Only		Combination Therapy	
		Count	%	Count	%	Count	%	Count	%	Count	%
N		4,054	100%	74	1.8%	2,364	58.3%	139	3.4%	1,477	36.4%
Mean Age at Diagnosis (years)		67		71		69		73		63	
Gender	Male	2,431	60.0%	49	66.2%	1,343	56.8%	93	66.9%	946	64.0%
	Female	1,623	40.0%	25	33.8%	1,021	43.2%	46	33.1%	531	36.0%
Race*	White	3,080	76.2%	46	63.0%	1,842	78.2%	98	70.5%	1,094	74.2%
	African American	298	7.4%	12	16.4%	150	6.4%	16	11.5%	120	8.1%
	Hispanic	356	8.8%	7	9.6%	190	8.1%	18	12.9%	141	9.6%
	Asian/Pacific Islander/Other	310	7.7%	8	11.0%	175	7.4%	7	5.0%	120	8.1%

Demographic data

The mean age at diagnosis of the cohort was 67 years. Patients receiving CSR were the youngest (63 years), followed by those undergoing surgery alone (69 years), no treatment (71 years), and radiation alone (73 years). Patients receiving CSR were significantly younger than those undergoing surgery alone (63 vs. 69 years, p<0.001).

Sixty percent of the cohort was male (N=2,431) and 40.0% (N=1,623) were female, resulting in a male to female ratio of 1.5:1, p<0.001. Among the patients receiving CSR, 64.0% were male (N=946) and 36.0% were female (N=531), resulting in a male to female ratio of 1.78:1, p<0.001. Among the patients undergoing surgery alone, 56.8% were male (N=1,343), and 43.2% were female (N=1,021), resulting in a male to female ratio of 1.32:1, p<0.001.

The majority of T2N0 rectal cancer cases occurred in Caucasians (N=3,080; 76.2%), followed by Hispanics (N=356; 8.8%), Asian/Pacific Islanders (N=310; 7.7%) and African Americans (N=298; 7.4%), p<0.001 (Table 1).

Tumor characteristics

 A majority of T2N0 rectal cancers were adenocarcinoma NOS (N=3,908; 96.4%), while 3.6% (N=145) were mucinous adenocarcinoma and <0.1% (N=1) were tubular adenocarcinoma. Similar trends were found in both patients receiving CSR and surgical resection alone.

Almost 6.2% (N=917) of T2N0 rectal cancers were > 4 cm in size, 58.6% (N=2,054) were 2-4 cm, and 15.2% (N=532) were < 2 cm, p<0.01. Patients receiving CSR had significantly more tumors < 2 cm (22.4% vs. 11.8%) and significantly less tumors between 2 cm and 4 cm (53.6% vs. 61.2%) and > 4 cm (24.0% vs. 27.0%) as compared to those treated with surgical resection alone, p<0.001.

Eighty-two percent (N=3,152) of T2N0 rectal cancers were moderately differentiated while 10.0% (N=382) were poorly differentiated, 7.3% (N=281) were well-differentiated, and 0.6% (N=23) were undifferentiated. Patients receiving CSR had significantly more moderately differentiated tumors (83.5% vs. 81.8%) and less poorly (9.3% vs. 10.1%) and undifferentiated (0.4% vs. 0.6%) tumors as compared to those treated with surgical resection alone, p<0.001 (Table 2).

**Table 2:** Tumor Characteristics of 4,054 Patients with T2N0 Rectal Cancer from the Surveillance, Epidemiology, and End Results (SEER) Database, 1973-2010 Abbreviations; cm. = centimeters; N = number; NOS = not otherwise specified; * data presented for patients with available information only

T2 NO Rectal Cancer		Total		No Therapy		Surgery Only		Radiation Only		Combination Therapy	
		Count	%	Count	%	Count	%	Count	%	Count	%
N		4,054	100%	74	1.8%	2,364	58.3%	139	3.4%	1,477	36.4%
Histology	Mucinous adenocarcinoma	145	3.6%	2	2.7%	74	3.1%	3	2.2%	66	4.5%
	Tubular adenocarcinoma	1	0.0%	0	0.0%	1	0.0%	0	0.0%	0	0.0%
	Adenocarcinoma, NOS	3,908	96.4%	72	97.3%	2,289	96.8%	136	97.8%	1,411	95.5%
Size*	Under 2 cm	532	15.2%	2	5.7%	263	11.8%	7	8.3%	260	22.4%
	2 to 4cm	2,054	58.6%	21	60.0%	1,361	61.2%	51	60.7%	621	53.6%
	Over 4 cm	917	26.2%	12	34.3%	601	27.0%	26	31.0%	278	24.0%
Grade*	Well differentiated	281	7.3%	5	8.2%	173	7.5%	11	10.0%	92	6.8%
	Moderately differentiated	3,152	82.1%	46	75.4%	1,892	81.8%	84	76.4%	1,130	83.5%
	Poorly differentiated	382	10.0%	10	16.4%	234	10.1%	12	10.9%	126	9.3%
	Undifferentiated	23	0.6%	0	0.0%	14	0.6%	3	2.7%	6	0.4%

Outcomes

Of all T2N0 rectal cancer patients, 58.3% (N=2,364) were treated with surgery alone while 36.4% (N=1,477) were treated with CSR. Of the patients, 3.4% (N=139) received radiation alone and 1.8% (N=74) received no treatment. Patients treated with CSR as the primary modality of treatment experienced significantly prolonged overall survival (5.85 ± 0.06 years) as compared to those who received surgical resection alone (5.44 ± 0.05 years), radiation alone (3.80 ± 0.27 years), and no treatment (2.31 ± 0.34 years), p<0.001. Patients receiving CSR had significantly longer mean survival times (5.85 ± 0.06 years vs. 5.44 ± 0.05 years, p<0.001) but a similar mean cancer-specific survival (6.33 ± 0.05 years vs. 6.42 ± 0.04 years, p=0.143) as compared to surgical resection alone.

**Figure 1:**
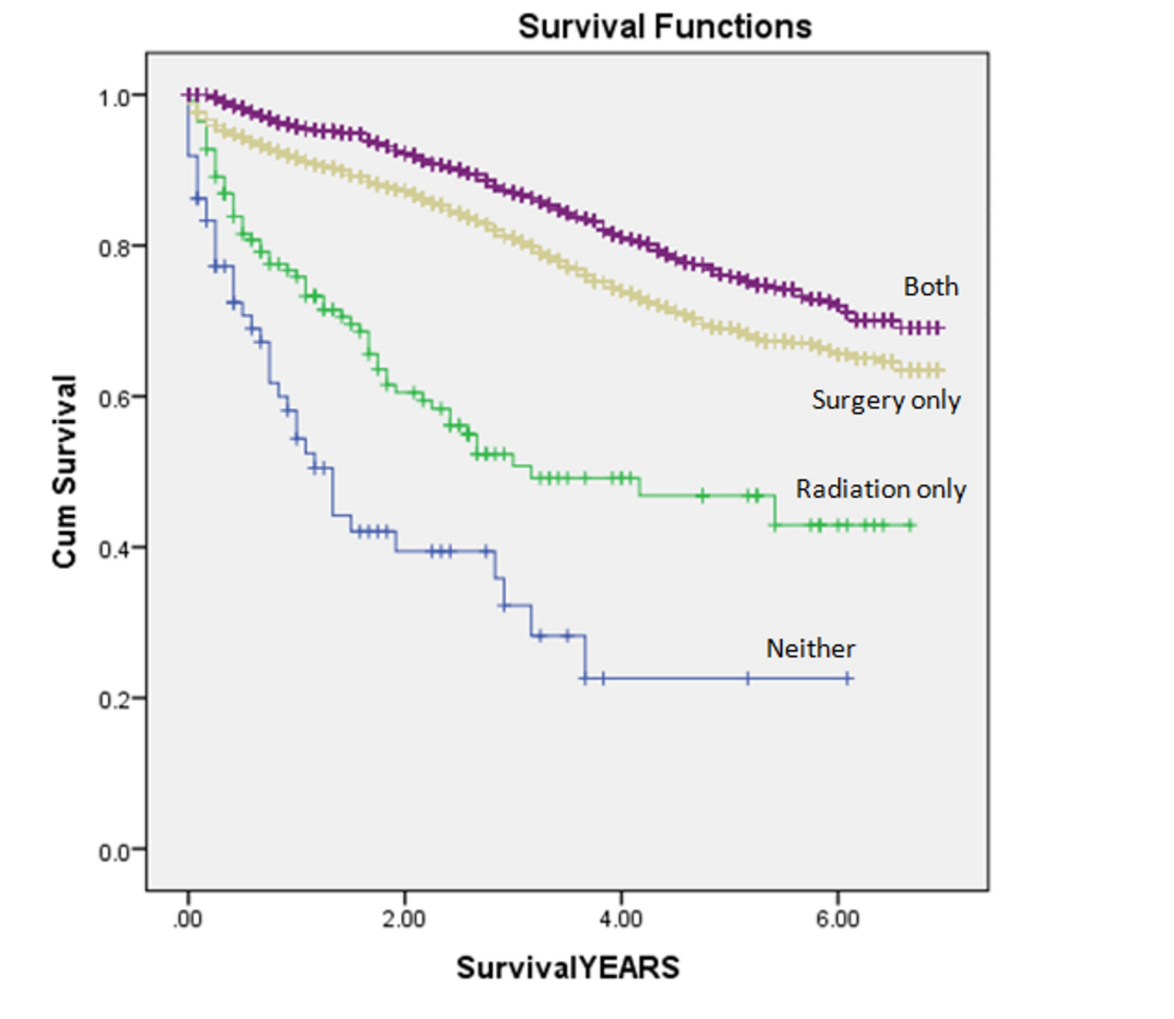
Kaplan-Meier curves Kaplan-Meier curves illustrating overall mortality for patients with T2N0 rectal cancer.

**Figure 2:**
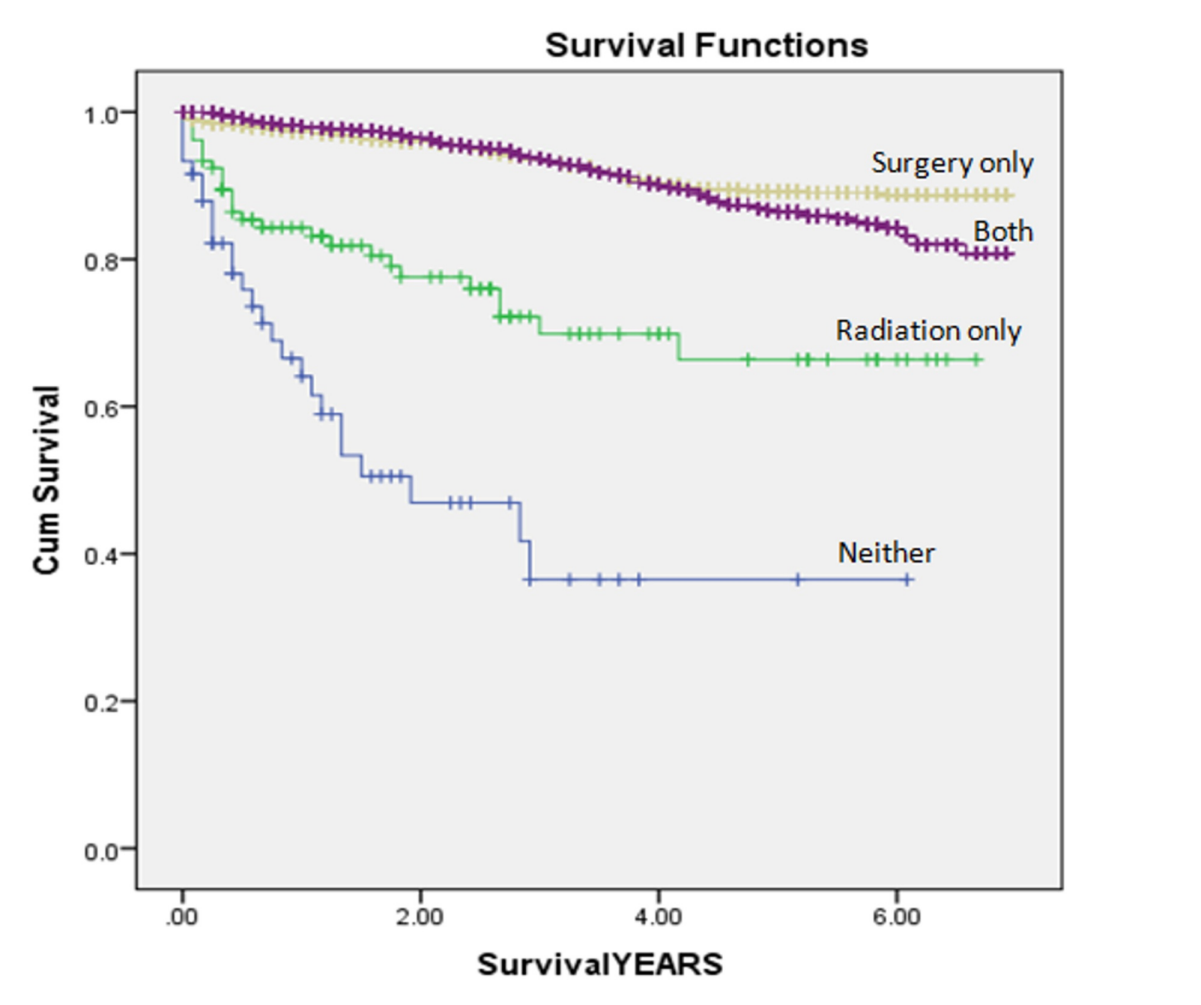
Kaplan-Meier curves Kaplan-Meier curves illustrating cancer-specific mortality from patients with T2N0 rectal cancer.

Patients treated with CSR experienced significantly lower overall mortality (16.3% vs. 22.6%) but higher cancer-specific mortality (8.7% vs. 7.3%) as compared to those receiving surgical resection alone (Table 3) (Figures 1-2).

**Table 3:** Treatment and Survival Outcomes of 4,054 Patients with T2N0 Rectal Cancer from the Surveillance, Epidemiology, and End Results (SEER) Database, 1973-2010 Abbreviations; N = number

T2 NO Rectal Cancer		Total		No Therapy		Surgery Only		Radiation Only		Combination Therapy	
Count	%	Count	%	Count	%	Count	%	Count	%
N		4,054	100%	74	1.8%	2,364	58.3%	139	3.4%	1,477	36.4%
Mean Overall Survival (years)		5.50±0.04		2.31±0.34		5.44±0.05		3.80±0.27		5.85±0.06	
Mean Cancer Specific Survival (years)		6.30±0.03		2.91±0.42		6.42±0.04		4.93±0.29		6.33±0.05	
Overall mortality	Alive	3,179	78.4%	34	45.9%	1,829	77.4%	80	57.6%	1,236	83.7%
	Dead	875	21.6%	40	54.1%	535	22.6%	59	42.4%	241	16.3%
Cancer specific mortality	Alive	3,179	91.0%	34	56.7%	1,829	92.7%	80	75.5%	1,236	91.3%
	Non cancer death	562	12.6%	14	10.7%	392	15.4%	33	17.9%	123	7.6%
	Cancer death	313	9.0%	26	43.3%	143	7.3%	26	24.5%	118	8.7%

Multivariate analysis

The multivariate analysis identified age > 60 (OR 2.4), poor (OR 1.7), undifferentiated grade (OR 2.6), and tumor size > 2cm (OR 1.5) as independently associated with increased mortality in the CSR group (p<0.05) while female gender conferred a survival advantage (OR 0.8), p<0.01.

## Discussion

T2N0 (Stage IB) rectal cancer is defined by tumor invasion into, but not through, the muscularis propria of the rectal wall and no nodal involvement (5). Based on the current study, T2N0 rectal cancer appears more prevalent amongst male Caucasians in their seventh decade of life, which is consistent with prior studies [5]. Early symptoms of rectal cancer include bleeding per rectum, a sense of incomplete defecation, and early morning diarrhea [6]. Histologically, rectal tumors are adenocarcinoma, originating from the glandular epithelium of the rectal mucosa [7]. Occasionally, the poorly differentiated histology of the tumor is also contributed to the excessive secretion of the mucous secondary to the mucinous adenocarcinoma [8].

Advances in radiological imaging have permitted better delineation and improved sensitivity in detecting Stage 1B rectal cancers [9]. Preoperatively, both endoscopic rectal ultrasound (ERUS) and magnetic resonance imaging (MRI) of the pelvis should be done in all rectal cancer patients for locoregional staging [10]. ERUS is the most sensitive diagnostic technique for differentiating T2 and T1 rectal cancers [11]. High-resolution MRI of the pelvis has higher accuracy than ERUS for the detection of mesorectal and local vascular infiltration in rectal tumors (T3 and T4 tumors) [12]. MRI also has the advantages of delineating the full extent of low-lying rectal tumors, more accurate measurement of tumor size, and providing information necessary to predict if tumor-free resection margins are present and other prognostic factors for the rectal tumors [13].

Surgical therapy with total mesorectal excision (TME) is the mainstay of the management for T2N0 rectal cancers offering excellent local control and resulting in superior long-term survival [14]. Studies have reported that a five-year local recurrence rate and disease-free survival (DFS) in T2N0 rectal cancer patients treated with TME is 3%-4.5% and 80%-90%, respectively [15]. However, some studies have reported a very high local recurrence (32%) following TME [16]. Additionally, due to the radical nature of the procedure, the morbidity of 30%-68% and a mortality rate of 5%-7% have been reported with the use of TME [16]. Also, this type of radical approach is associated with significant complications, including sexual dysfunctions (30%-40%), bowel and bladder dysfunctions (30%-40%), depression (30%), anastomotic leakage (5%-17%), and a possible permanent colostomy in 40% of patients [17-18]. Given the high morbidity associated with TME, alternative approaches to the management of T2N0 rectal cancers have been investigated. In a literature review on the management of early-stage rectal cancers, Stamos et al. (2007) demonstrated a significant rise in the use of transanal excision for T2 rectal cancers (44%) between 1989 and 2003. Yet, a constant high rate of locoregional recurrences of disease after this procedure (47%) tumors was observed, suggesting that LE and adjuvant radiation therapy might have a role to play in the management of T2N0 rectal cancers [19].

The use of local excision (LE) with adjuvant pelvic radiation in carefully selected patients with T2N0 rectal cancer has been used as an alternative to the traditional radical approach [19]. Duek et al. reported that transanal endoscopic microsurgery with adjuvant radiation for the treatment of selected patients with T2 rectal cancers could be a possible alternative to the more radical approach [20]. Of the 21 patients diagnosed with T2N0 rectal cancers between 1995 and 2005, 12 agreed to receive local excision and adjuvant radiation therapy [20]. The majority of the patients undergoing adjuvant radiation were female, with a mean age diagnosis of 68 years [20]. Most of the tumors had clear margins, were well-differentiated to moderately differentiated, located in the posterior rectal wall, were 8.4 cm from the dentate line, and had an average tumor size of 2.7 cm [20]. The morbidity in this cohort was minimal, and deaths related to the surgical procedures were reported [20]. At the three years follow-up, the group of patients who received both radiotherapy and local excision was complete disease-free while the group who refused radiotherapy had a recurrence rate of almost 50% [20]. Additionally, no local recurrence was noticed in all patients treated with adjuvant radiation at a follow-up of 58 months [20].

Another study conducted by Minsky et al. reported that in very carefully selected rectal cancer patients with no evidence of gross tumor in the pelvis, local excision with pelvic radiation might be used as an alternative to the more radical approach [21]. They observed that all T2 rectal tumors with 28% penetration of the rectal wall, with moderately and poorly differentiated grade, total colloid (mucinous) histology, blood vessel involvement (BVI), and lymphatic vessel involvement (LVI) show an incidence of lymph node positivity (LN ) by ≥19%. The study concluded that if there is an absence of the tumor expansion into the pelvis, the radical surgery approach can be switched with a combination of the adjuvant radiation directed towards the pelvis and local excision [21].

Also, Lezoche et al. (2005) emphasized the critical role of adjuvant radiotherapy in the local control of highly selected Stage 1B rectal cancers [22]. In their study, they reported that when radiation is combined with LE for the highly selected group of Stage 1B rectal cancers, the local recurrence (15%), disease-specific relapse at distant organs (10%), and overall survival at 5 years (80%) were comparable to those observed after the radical procedure [22]. Another study conducted by Stitzenberg et al. reported that those Stage 1 rectal cancer patients who were treated by LE (OR, 4.9; 95% of the CI (4.5-5.3), those who have T2 tumors (OR, 4.1; 95% of the CI, 3.8- 4.5), those with high-grade tumors, and those with positive margins (OR, 3.1; 95% of the CI, 2.7- 3.5) were most commonly the candidates to get the adjuvant radiation therapy [23].

Also, Rich et al. studied 26 patients with Stages T1, T2, and T3 rectal cancers who refused more radical approach and underwent LE with pelvic radiation and reported that this approach proved to be a safe alternative option to the dangerous radical surgery in carefully selected patients and resulted in excellent local control [24]. In the 17 patients with no gross residual tumor, there was a 94% local control rate (16 of 17) and the disease-free survival rate was 88% [24].

Additionally, the oncological outcomes in carefully selected early rectal cancer patients treated with local excision and adjuvant radiotherapy are similar to TME. In a systematic review of 11 studies comprising 450 patients with T1/T2N0M0 rectal cancer and receiving local excision with adjuvant therapy, Ung et al. reported that in this group, the overall survival (OS), the disease-specific, and the disease-free survival was 75%, 89%, and 74%, respectively. They also reported that local, distant, and overall recurrence in this group was 10%, 4.7%, and 13.1%, respectively [25]. They compared their results with the outcomes of the patients who received the TME alone in the Dutch Colorectal Cancer Trial. After the comparison, they found that there was little difference in OS, local recurrence, and overall recurrences at two years (81.8%, 8.2%, and 20.9%) [25].

Also, Stitzenberg et al. in a study to examine the updated patterns of the practice and overall survival rates in patients diagnosed with the early-stage rectal cancers observed the OS for those T2N0 cancer patients who had radical surgery alone, radical surgery and adjuvant radiation and LE plus adjuvant radiation. Their study reported that OS for T2N0 rectal tumors treated with LE and adjuvant radiotherapy was similar to survival for the comparable group treated by radical surgery and radical surgery with adjuvant radiation [23]. Furthermore, a study conducted by Fortunato et al. for the long-term follow-up of local excision and adjuvant radiation therapy reported that this treatment approach could be offered to those highly selected T2 rectal cancer patients who refuse a more radical approach due to the risk of a permanent colostomy or are they medically compromised [26]. In their study, two patients had T1 tumors, 15 had T2 tumors, and four patients had T3 tumors. The median size of the tumor in their study was 3 cm, and the distance from the anal verge was a median of 4 cm. They found that five years overall, disease-free, and recurrence-free survival was 77%, 75%, and 58% [26].

Furthermore, the review of literature explains that even with the most radical approach, the risk of recurrence for T2N0 rectal cancer remains high; the recurrence rate increases as the distance of the tumor from anal cancer decreases and rate reaches 16.2% in distal rectal tumors [27]. The higher recurrence rate for the distal rectal tumors provides evidence that LE with adjuvant radiation would be most valuable for improving the outcome in this group [28].

One major issue with the use of adjuvant radiation is whether the patients receiving adjuvant radiotherapy are compromised by the toxicity of the treatment or not. Edwards et al. reported that adjuvant radiation treatment increases the healing probability after the surgery [29]. Also, a review of the literature on the side effects of radiation in rectal cancer patients demonstrates that radiotherapy is well-tolerated by rectal cancer patients, especially in neoadjuvant settings [29]. However, the question of how much treatment-related toxicity is added with adjuvant radiation therapy can only be answered after multiple prospective trials analyzing the appropriate dose, timing, method of delivery, and side effect of adjuvant radiation in properly selected T2N0 rectal cancer patients are conducted.

In the current study, combined surgery and radiation (CSR) was utilized primarily in younger male Caucasians presenting with limited locally advanced disease compared to other treatment groups. Although it prolonged overall survival, cancer-specific mortality was not improved, implying that the observed improvements in overall survival may be more attributable to favorable non-tumor-related factors such as age, gender, and race. A possible explanation for the CSR in the younger patients maybe patient-driven, with more patients preferring therapies with minimal impact on the quality of life [30]. As CSR is the less aggressive mode of treatment compared to conventional radical approach, the lower morbidity associated with the CSR may explain the prolonged overall survival and lower mortality related to the use of CSR [30].

There are several limitations to this study that should be considered. First, the SEER database does not correctly code for all critical clinical factors, which might contribute to the additional cohort related information. These factors include but are not limited to socioeconomic status, geographical location, and the precise depth of the mass. Second, the information related to the diagnostic confirmation and the proper follow up period is also lacking in the SEER database. Also, the precise stage-specific surgical procedure was not available in the SEER database. Another limitation of the current study is the location of the tumor in different parts of the rectum. Additionally, information regarding the dose and side effects of the adjuvant radiation cannot be extracted from the SEER database.

## Conclusions

Currently, there is no clear consensus on the best course of management for T2N0 rectal cancers and studies are limited. While surgery remains the standard method of treatment for Stage 1B rectal cancer, adjuvant radiation has been becoming more promising. The current study represents the largest cohort of T2N0 rectal cancer patients to date, establishing trends in demographics and clinical outcomes in a large US patient cohort receiving adjuvant radiation. In this cohort, adjuvant radiotherapy was utilized most often in young male Caucasian patients presenting with less locally advanced disease as compared to other treatment groups. Adjuvant radiation significantly prolongs overall survival and decrease overall mortality in T2N0 rectal cancer. Although the use of adjuvant radiotherapy prolonged overall survival, the lack of cancer-specific survival benefit and increased cancer-specific mortality in the adjuvant radiation group imply that more prolonged overall survival observed may be attributable to more favorable non-tumor-related factors such as age, gender, and race. While current guidelines for diagnosing and treating T2N0 remains unchanged, adjuvant radiation should not replace surgery alone as the standard of care for all Stage IB rectal cancer patients at this time. However, all T2N0 rectal cancer patients should be enrolled in randomized control trials or registries to allow for more defined multimodality management to optimize clinical outcomes for these patients.
